# Task Integration Facilitates Multitasking

**DOI:** 10.3389/fpsyg.2017.00398

**Published:** 2017-03-15

**Authors:** Rita F. de Oliveira, Markus Raab, Mathias Hegele, Jörg Schorer

**Affiliations:** ^1^School of Applied Sciences, London South Bank UniversityLondon, UK; ^2^Institute of Psychology, German Sport University CologneCologne, Germany; ^3^Neuromotor Behavior Laboratory, Giessen UniversityGiessen, Germany; ^4^Institute of Sport Science, University of OldenburgOldenburg, Germany

**Keywords:** multitasking, structure, pursuit tracking, attention, implicit learning, dual-task

## Abstract

The aim of this study was to investigate multi-task integration in a continuous tracking task. We were particularly interested in how manipulating task structure in a dual-task situation affects learning of a constant segment embedded in a pursuit-tracking task. Importantly, we examined if dual-task effects could be attributed to task integration by varying the structural similarity and difficulty of the primary and secondary tasks. In Experiment 1 participants performed a pursuit tracking task while counting high-pitched tones and ignoring low-pitched tones. The tones were either presented randomly or structurally 250 ms before each tracking turn. Experiment 2 increased the motor load of the secondary tasks by asking participants to tap their feet to the tones. Experiment 3 further increased motor load of the primary task by increasing its speed and having participants tracking with their non-dominant hand. The results show that dual-task interference can be moderated by secondary task conditions that match the structure of the primary task. Therefore our results support proposals of task integration in continuous tracking paradigms. We conclude that multi-tasking is not always detrimental for motor learning but can be facilitated through task-integration.

## Introduction

Multi-tasking is known to have detrimental effects on learning and most researchers agree that resource allocation is a major factor. The argument is that two tasks sharing resources compete for their allocation and thus interfere with each other if executed at the same time; this also impairs skill learning (Eimer et al., 1996). One example of interference was shown in the study of Nissen and Bullemer (1987). Sequence learning was impaired in a serial reaction time task (SRT task) done in conjunction with a secondary tone-counting task. The proposed explanation was that the impairment was due to the high capacity load of the secondary task (see also Curran and Keele, 1993). Other researchers have studied how the characteristics of the primary SRT task may influence interference (Hsiao and Reber, 1998), for example: the sequence length (Sanchez and Reber, 2012); the overall stimulus statistics beyond event-to-event co-variations (e.g., Stadler, 1995); and the structure of the sequence (e.g., Cohen et al., 1990; Reed and Johnson, 1994). There is currently no agreement as to whether dual-task interference may be reduced through task integration. For example Heuer and Schmidtke (1996) proposed that dual-task interference may be reduced under circumstances that allow for task integration while Ruthruff et al. (2006) found no evidence for task integration. Most of the dual-task literature to date has used the SRT paradigm which requires the response to two discrete tasks. By using one continuous tracking task and one discrete task we can investigate dual-task integration strategies where task integration is both possible and potentially beneficial to learning and performance.

Recently three main contributing factors to reduce dual-task costs in SRT paradigms have been proposed: difficulty of the tasks, structural similarity between the tasks, and practice (e.g., Hazeltine et al., 2002; Huang and Pashler, 2005). These are the three factors that will constitute the manipulations in the present study. Difficulty of the tasks increases interference because the demands on the cognitive and motor system increase when better action coordination is required. Several explanations have been presented as to the exact mechanism underlying the impairing effects of increased task difficulty (Wickens, 1980; Ruthruff et al., 2006). There is a general agreement that more difficult tasks require more attentional resources in order to be executed, and therefore in a dual-task situation there will be fewer resources available to complete another task. In this connection, the order of task execution (i.e., whether the harder task is primary or secondary) may be of relevance. Ruthruff et al. (2006) conducted two experiments in which participants practiced only an audio-vocal task 1, practiced only a visual-manual task 2 or both as a dual-task. They argued that according to the task integration hypothesis dual-task practice would be more effective than single task practice in reducing or eliminating the central bottleneck believed to cause dual-task interference. In contrast, the automatization hypothesis predicts elimination of the bottleneck independently of whether single or dual-task conditions have been practiced. Finally the intact bottleneck hypothesis predicts that effects should be smaller after dual-task or task 1 practice in comparison to task 2 practice. Their results point to an intact bottleneck in Experiment 1 and to automatization of one or both tasks in Experiment 2, which is why the authors interpreted results as supporting automatization.

Practice is thought to reduce interference through increased automaticity (Schneider and Shiffrin, 1977; Ruthruff et al., 2006). Practice is also crucial for the decreased attentional load during implicit learning. In implicit learning paradigms, people acquire the skills unintentionally and without awareness of what is being learned (Hsiao and Reber, 1998; Magill, 1998; Masters, 2000; Jiménez, 2003; Sun et al., 2005; Kleynen et al., 2014, for reviews). While it is well known that attention to movement coordination hampers performance (Hossner and Wenderoth, 2007; Wulf, 2007) less is understood about the beneficial effects of implicit learning on dual-tasks (i.e., two tasks learned implicitly and simultaneously). However, it has been suggested that smaller attentional demands in implicit learning should benefit dual-task learning and performance (see Berry and Dienes, 1993, for an overview; Beilock and Carr, 2001; Maxwell et al., 2003; Franklin et al., 2016). Most studies have used a dual-task paradigm to test whether implicitly learned movements can be produced more reliably under dual-task conditions, than explicitly learned movements (Hsiao and Reber, 1998, for an overview). However, these studies used relatively simple reaction-time tasks, and not much is known about dual-task effects when more complex tasks are carried out simultaneously. An example of complex multi-tasking that we still know little about is driving, which involves steering, pedals control (de Oliveira and Wann, 2011) but also the control of indicators, windscreen wipers and other devices. In a recent study, Huestegge and Koch (2014) demonstrated that in strongly automatized dual-tasks such as eye-movement and manual key press there is a cost associated with saccade inhibition. The authors propose that this is a case where a single task (key press) is harder than a dual-task (saccade with key press) although one can instead propose that the half-task (key press) is harder than the single task (saccade with key press). Regardless, this study shows that highly automatized tasks should be regarded as a unit because of the extra costs inhibiting part of that unit.

The structural similarity between the primary and secondary tasks may mean that the two tasks can be integrated. This would reduce interference because similar features of the two tasks can be temporally integrated into one task (Heuer and Schmidtke, 1996). The authors suggest a situation where one task consists of responding manually to visual stimuli which appear every 200 ms in a certain sequence (visual-manual), and a second task consists of counting only the high-pitched tones which appear randomly every 200 ms in the interval between visual stimuli (audio). After learning the single visual-manual task, performance in a dual-task would be poor because, Heuer and Schmidtke (1996) suggest, this would be a new sequence with alternating visual-motor and audio stimuli. In a subsequent study, Schmidtke and Heuer (1997) tested whether such intermodal integration could explain dual-task interference. First they found evidence of dual-task integration as hypothesized, but more importantly they found that dual-task integration could be beneficial to performance. When the tone sequence and the visual stimuli sequence had the same number of elements, their integration was easier and dual-task performance was better than when the tone sequence had a different number of elements. Interestingly, when a sequence was presented but no response was required, this effect was no longer present. This means that the beneficial effects of task integration may only be visible when participants actively engage with learning it. These results are in accordance with other authors who also found that structural similarity between the tasks allowed a consistent organization of the SRT task sequence which benefitted performance (Stadler, 1995; Stadler and Neely, 1997; Dominey, 1998). They also support the view that the exploration of co-variations in environmental stimuli is a fundamental operation of our perceptual-motor system (Gibson, 1979; Reber, 1989).

Although most dual-task research has been conducted under the SRT paradigm, pursuit-tracking may offer another perspective to examine both implicit visual-motor control and the hypothesis of task integration. Importantly, pursuit tracking requires continuous resource allocation for the continuous visual guidance of movements (Poulton, 1952; Pew, 1974). It also allows implicit motor learning to occur by embedding a constant movement pattern within a randomly generated tracking pattern. It has been shown that a repeated middle segment is learned better than outer pseudorandom segments even though participants are not aware of the repetition (Poulton, 1952). These findings have been replicated by several research groups (Magill and Hall, 1989; Wulf et al., 1994; Wulf and Schmidt, 1997; Magill, 1998; Shea et al., 2001, but see Chambaron et al., 2006). Still, no systematic research program relating attention to complex implicit motor learning has been established. Using a pursuit-tracking task we can examine whether the possibility of task-integration facilitate or hamper performance in multi-tasking.

The aim of this study is to investigate multi-task integration in implicit motor learning and performance. We use a pursuit-tracking paradigm as a primary task and a response to high- and low-pitched tones as secondary tasks. In Experiment 1 we implement a structural similarity condition between the primary and secondary task by presenting the high-pitched tones 250 ms before each reversal point. We expect performance to be better than when the tones appear randomly and this would support the hypothesis that the tasks were learned and performed in an integrated manner. In Experiments 2 and 3 we increase the motor load of the secondary and primary tasks, respectively, to test the robustness of task integration (see **Table 1**).

**Table 1:** Study design over the three experiments including primary task, secondary task and group manipulations.

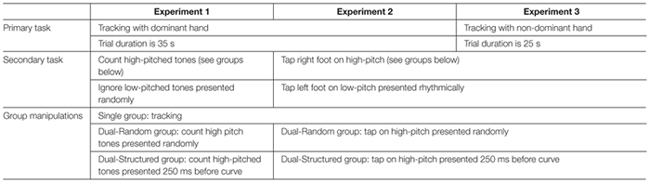

*See further details in the Methods sections of the respective experiments.*

## Experiment 1

The goal of the first experiment was to test the use of task integration in a visuomotor tracking task. We asked participants to perform a manual tracking task while counting high-pitched tones and ignoring low-pitched tones. In order to explore the effects of task integration, we created three experimental conditions consisting of a Single group who performed the tracking task only, a Random group for whom the low- and high-pitched tones were presented randomly, and a Structure group for whom the low-pitched tones were random but the high-pitched tones were temporally coupled to events in the tracking task. Tone counting was chosen as a secondary task as this task has been used in most dual-task SRT studies. We hypothesized that the Structure group out-perform the Random group because the structure of the secondary task allows for task integration in implicit motor learning.

### Method

#### Participants

Participants were 30 university students (14 female and 16 male; *M* = 26.8 years, *SD* = 3.4; 6 left-handed and 24 right-handed). They had normal or corrected-to-normal vision, normal hearing abilities and no prior experience in tracking. Participants were randomly assigned to three groups of 10 with similar distribution in gender and lateralization. Ethics approval for the study was obtained from the Institute for Sport and Sport Science of the University of Heidelberg, Germany. Participants provided informed consent before starting the experiment.

#### Apparatus and Material

A pursuit tracking task was adapted from Magill and Hall (1989). Participants followed a red target cross (∼1.5 cm) on a black screen (800 × 600 pixels) with a white control cross (∼1 cm), manipulated via a computer mouse with their self-reported dominant hand. The mouse was controlled in both the *x* and *y* directions. For the primary task, the target cross consisted of a constant middle and two pseudorandom outer segments. The baseline performance of the three segments was pilot-tested. Varied amplitudes have shown differences in performance (Magill, 1998), therefore we used the same mean amplitude for the three segments. The tracking curve of the constant segment was derived by f(x) = sin (x) × cos (x × 2.41) + sin (x × 2.1) × cos (x × 2.7) and the pseudorandom segments by f(x) = pai × sin (x × i) + pbi × cos (x × i) as described by Wulf and Schmidt (1997). Equations for the three segments were graphic representations of a trigonometric series in which indices refer to coefficients of the polynomial. The values for the indices were selected such that the index of tracking difficulty (Wickens, 1980) was identical for all segments and that smooth transitions between segments could be used. We pilot-tested different speeds and set the trial duration at 35 s.

For the secondary tasks we used high-pitched and low-pitched tones (respectively, 1,086 Hz, 52 ms and 217 Hz, 350 ms, see Hsiao and Reber, 1998). Participants were instructed to count the high-pitched tones, ignore low-pitched tones and to recall the total number of high-pitched tones at the end of each trial. High-pitched tones were coupled to the structure of the tracking task such that they were presented 250 ms before each reversal point of the curve or randomly distributed at various locations along the curve. Low-pitched tones were presented randomly along the curve. There were a similar number of high- and low-pitched tones per trial each ranging between 12 and 18.

#### Design

We used a factorial design with three factors: Group × Block × Segment. There were three groups: a Single group who only performed the primary tracking task; a Random group for who the low- and high-pitched tones were presented randomly; and a Structure group for who the low-pitched tones were random but the high-pitched tones were presented 250 ms before each curve. There were six blocks of acquisition with 40 trials each, a group-retention test (i.e., retention test under group practice conditions), and a *x*-transfer test (i.e., the middle segment was reversed along the *x*-axis). There were two types of segment: the middle constant segment and the pseudorandom outer segments (Raab et al., 2013).

#### Procedure

Participants reported to the laboratory for the experimental sessions on three consecutive days (two participants did it in four consecutive days because of scheduling issues). Upon arrival on the first day, participants were given written instructions to follow the red cross with the mouse-controlled white control cross. Participants sat approximately 50 cm in front of a 17-inch monitor, in a dim and quiet room equipped with stereo headphones (used in the multi-task groups). The written instructions encouraged participants to minimize the tracking error (i.e., the root mean square error or RMSE; Single group), or to minimize both the tracking error and the tone-counting errors (Random and Structure groups) with equal emphasis (Ruthruff et al., 2003).

The experiment started with a first block of 40-trial warm-up phase in which the participants got used to their group-specific condition (see **Table 2** for study schedule). On the first 2 days participants performed three blocks of 40 trials for a total of 240 acquisition trials. At the end of each trial, participants in the Random and Structure conditions were asked to type the number of high-pitched tones they had counted. Then participants received feedback regarding their tracking error in terms of RMSE and their tone-counting performance. After Day 1, participants answered a questionnaire concerning personal information, instruction check, and motivation level. To test potential moderators, it also included questions regarding experience in controlling a mouse, playing computer games and instruments, and doing two things at once (Joseph and Willingham, 2000). On Day 3, participants performed a group-specific retention test. Participants then performed a group-specific transfer test in which the middle segment was reversed along the *x*-axis. Afterwards participants answered a free-recall questionnaire regarding explicit knowledge of the constant segment with questions progressively more specific. They started with: “Did you notice anything about the task?” and ended with: “Did you notice that one segment was repeated over all trials?” and “Which segment was the repeated one?” On the last question participants were required to choose one of the three segments.

**Table 2:** Study schedule over the three experiments.

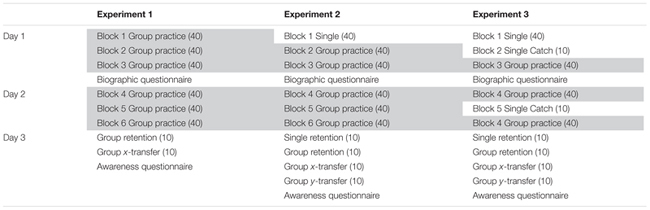

**Group* means the trials were done in the group-specific conditions; *Single* means the trials were done as a single task by all groups; *Catch* means the middle segment was pseudorandom (i.e., not constant). In parentheses is the number of trials in each block. Group practice for each experiment is shaded in light-grey. In *x*- and *y*- transfers the middle segment was reversed along the *x*- or the *y*-axis. Participants completed the experiments within 3 consecutive days.*

#### Data Analysis

The main dependent measure was the RMSE which is the difference between the path presented on the display and the path tracked by the participant. To test for acquisition, we submitted RMSEs to a Group × Block × Segment analysis of variance (ANOVA) with three levels on Group (Single vs. Random vs. Structure), two levels on Block (block 2 vs. block 6), and two levels on Segment (constant vs. pseudorandom). We also performed additional analyses for group-specific retention and transfer effects. To test for retention and transfer effects, we performed two Group × Block × Segment ANOVAs with three levels on Group, two levels on Block (to test retention: block 6 vs. group-retention; to test transfer: block 6 vs. group-transfer) and two levels on Segment. We used one-way ANOVAs or *t*-tests to investigate significant group effects where appropriate. Performance on the secondary task was assessed by calculating the percentage of correct high-pitched tone recalling and submitting those to independent *t*-tests. Prior to testing the main hypothesis, we found that there were no significant differences between the three groups in age, gender, previous experience in tracking, motivation, or instruction. Significance level was set at *p* < 0.05, data were tested for distribution normality, and where appropriate degrees of freedom were adjusted for violations of sphericity using the Huynh–Feldt correction. Partial eta squared is reported (small effects $\eta_{p}^{2}$ > 0.01; medium effects $\eta_{p}^{2}$ > 0.06; large effects $\eta_{p}^{2}$ > 0.14). Please note that RMSE measures are in arbitrary units.

### Results

#### Acquisition

**FIGURE 1:**
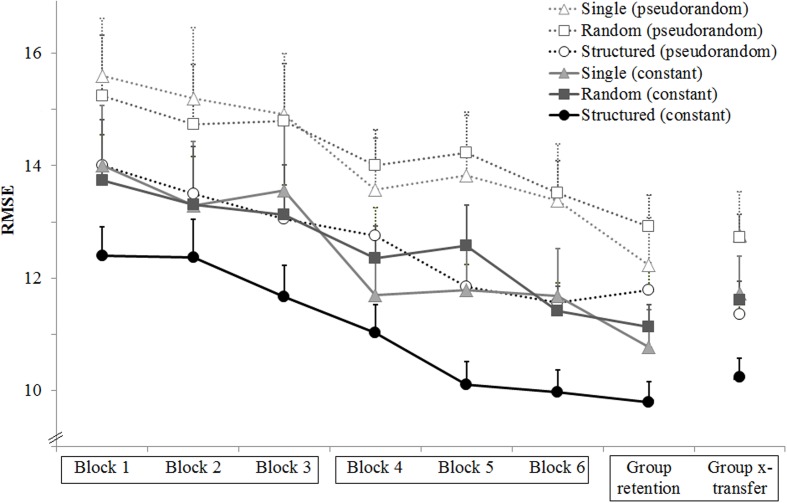
**Experiment 1: Mean RMSE over blocks, groups and segments.** Error bars are standard error of the mean. The *x*-labels are boxed according to the days when they were completed; Blocks 1–3 were completed in day 1, blocks 4–6 were completed in day 2, and the retention and transfer blocks were completed in day 3. All blocks were performed in the group-specific conditions.

There was a significant main effect of block, *F*(1,27) = 16.48, *p* < 0.05, $\eta_{p}^{2}$ = 0.38, indicating that overall tracking error was smaller in block 6 than in block 2 (respectively *M* = 11.9, *SD* = 2.2 and *M* = 13.7, *SD* = 3.3). There was a significant main effect of segment, *F*(1,27) = 313.66, *p* < 0.05, $\eta_{p}^{2}$ = 0.92, because mean error was smaller on the constant than the pseudorandom segment (respectively *M* = 12.0, *SD* = 2.2 and *M* = 13.7, *SD* = 2.2). There was no main effect of group, *F*(2,27) = 1.33, *p* > 0.05. Importantly, there was a Block × Segment interaction, *F*(1,27) = 5.17, *p* < 0.05, $\eta_{p}^{2}$ = 0.16, because from block 2 to 6 there was a larger improvement in the constant segment (difference of 2.0) than in the pseudorandom segment (difference of 1.7). There was also a Group × Block × Segment interaction, *F*(1,27) = 4.14, *p* < 0.05, $\eta_{p}^{2}$ = 0.24. This interaction occurred because the largest improvement was by the Structured group in the constant segment (difference of 2.4), whereas the smallest improvement was by the Random group in the pseudorandom segment (difference of 1.2; see **Figure 1**). A comparison showed that at block 6 the Structured group was significantly more accurate than the Single group in the constant segment, *p* < 0.05, and tended to be more accurate than the Single and Random groups on the pseudorandom segment, respectively *p* = 0.07, *p* = 0.06. No significant differences were found at block 2.

**FIGURE 2:**
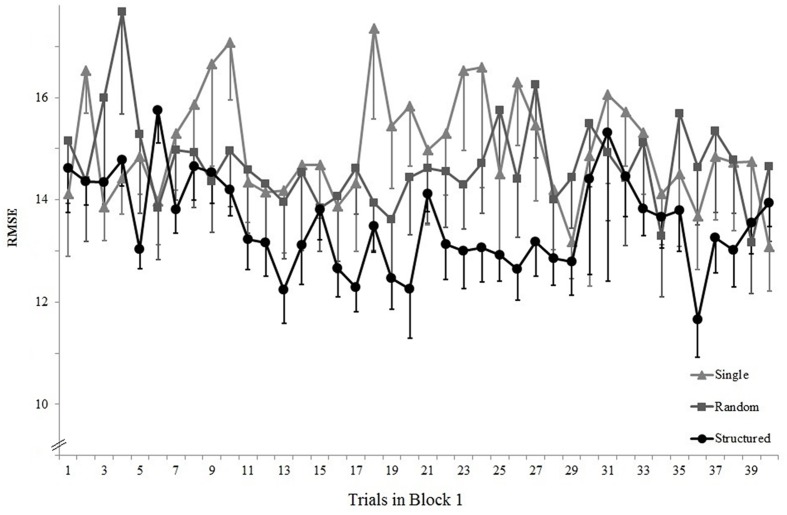
**Experiment 1: Mean RMSE for all trials of block 1.** Note that the Structure group starts showing smaller RMSE than the Single and Random groups after the 10th trial. Error bars are standard error of the mean.

To allay concerns that the group differences might reflect random pre-group differences already present before the start of the experiment, **Figure 2** shows all trials of Block 1 where it can be seen that the Structured group starts performing better than the other two groups after 10 trials.

#### Retention

There was a significant effect of block, *F*(1,27) = 6.00, *p* < 0.05, $\eta_{p}^{2}$ = 0.18, because mean error continued to decrease from block 6 to group-retention (difference of 0.5). There was a significant main effect of segment, *F*(1,27) = 195.65, *p* < 0.05, $\eta_{p}^{2}$ = 0.88, because mean error was smaller in the constant than the pseudorandom segment (difference of 1.8). There was no main effect of group, *F*(2,27) = 1.99, *p* > 0.05, and no significant interactions.

#### Transfer

There was no significant effect of block, *F*(1,27) = 0.84, *p* > 0.05. The significant effect of segment, *F*(1,27) = 221.48, *p* < 0.05, $\eta_{p}^{2}$ = 0.89, was caused by smaller mean error in the constant than in the pseudorandom segment (difference of 1.4). There was a tendency for a group effect, *F*(1,27) = 2.89, *p* = 0.07, $\eta_{p}^{2}$ = 0.18. Planned comparisons showed that the Structure group performed better than both the Single group (difference of 1.6, *p* < 0.05) and the Random group (difference of 1.5, *p* = 0.06). There was a significant Block × Segment interaction, *F*(1,27) = 17.59, *p* < 0.05, $\eta_{p}^{2}$ = 0.40, because from block 6 to transfer, there was a small improvement in the pseudorandom segment but not in the constant segment. There was no significant Group × Block × Segment interaction, *F*(1,27) = 1.54.

#### Secondary Task Accuracy

Participants in the Dual groups performed the secondary task well, with both groups in all blocks recalling between 91 and 100% of the high-pitched tones (overall *M* = 0.97, *SD* = 0.01). Group comparisons revealed group differences in block 3, *t*(18) = 2.16, *p* < 0.05 (*M* = 0.96, *SD* = 0.02 vs. *M* = 0.98, *SD* = 0.02) and block 6, *t*(18) = 2.61, *p* < 0.05 (*M* = 0.97, *SD* = 0.02 vs. *M* = 0.98, *SD* = 0.01) with the Random group showing better performance than the Structure group.

#### Explicit Knowledge

Participants did not verbalize knowledge or awareness of the constant segment. Asked to guess which of the three segments was repeated, 17 participants did not answer, 7 guessed incorrectly it was one of the outer segments, and 6 guessed correctly it was the middle segment (3 in the Single group, 1 in the Structure group and 2 in the Random group).

### Discussion

Experiment 1 supported our hypothesis; the Structure group outperformed both the Random group and the Single group after a small amount of practice. To recall, both multi-task groups counted the high-pitched tones while ignoring the low-pitched tones, but high-pitched tones were presented randomly to the Random group whereas they were presented 250 ms before each curve to the Structure group. Therefore we can propose that the shared structure between the primary and the secondary task contributed to the better performance in the Structure group. Interestingly, the superior tracking performance in the Structure group, while being particularly emphasized in the constant segment, also tended to manifest itself in the random segments indicating that cross-dimensional associations can facilitate performance even in non-predictable environments. This is an important result because it questions the suggestion that dual- or multi-tasking always hampers performance (but see Huestegge and Koch, 2014 who agree this is not always the case). The timing of the high-pitch presentation in the Structure group may have allowed for optimal coupling between the visuomotor information, the audiomotor information, and the motor action because previous research indicates an average delay of about 300 ms in tracking (de Oliveira and Wann, 2010). It seems that the tracking task became easier in the Structure group because each curve came with a (auditory) warning sign and perhaps also because participants may have counted curves as they turned them. This would effectively integrate the two tasks into one easier task. To investigate this further, we increased the motor requirements of the secondary task in Experiment 2.

## Experiment 2

The goal of the second experiment was to test the robustness of task integration by increasing the motor load of the secondary tasks. We asked participants to perform a manual tracking task while tapping a foot on high-pitched tones and tapping another foot on low-pitched tones (cf. Heuer and Schmidtke, 1996). The manipulation consisted of a Single group who performed the tracking task only, a Random group for who the low-pitched tones were presented rhythmically and the high-pitched tones were presented randomly, and a Structure group for who the low-pitched tones were also presented rhythmically but the high-pitched tones were temporally coupled to the tracking task. Again, we hypothesized that the Structure group would show out-perform the Random group because the structure of the secondary task allows for task integration.

### Method

#### Participants

Participants were 30 university participants (14 female and 16 male, *M* = 24.2 years, *SD* = 6.7; all right-handed). They had normal or corrected-to-normal vision, normal hearing abilities and no prior experience in tracking. Participants received course credit for participation and provided informed consent before starting the experiment. They were randomly assigned to one of the three groups.

#### Apparatus and Material

We used the same tracking task and the same high- and low-pitched tones as in Experiment 1. In this experiment participants responded by tapping their right foot on a Marquardt drum pad for high-pitched tones and by tapping their left foot on another Marquardt drum pad for low-pitched tones. High-pitched tones were coupled to the structure of the tracking task such that they were presented 250 ms before each extreme of the curve (Structure) or randomly distributed at various locations along the curve (Random). For both dual groups, low-pitched tones were presented rhythmically every 500 ms.

#### Design and Procedure

We used a factorial design with three factors: Group × Block × Segment. The groups were: Single, who only performed the primary tracking task; Random, who responded to low- and high-pitched tones presented randomly in relation to the curve; Structure, who responded to randomly presented low-pitched tones and high-pitched tones presented 250 ms before each curve.

Participants reported to the laboratory for the experimental sessions on three consecutive days. During acquisition on each of the first 2 days participants completed three blocks of 40 trials each for a total of 240 trials. The first of the six blocks was under a single-task condition and was used as a baseline. For the subsequent five blocks participants performed group-specific tasks (i.e., single, random or structure; see **Table 2**). At the end of day 1, participants answered the same questionnaire as in Experiment 1 concerning possible moderators. On day 3, participants completed a retention test of 10 trials in single-task, followed by another 10 retention trials in their group-specific condition. This was followed by two transfer tasks of 10 trials each, with the middle segment reversed along the *x*-axis or the *y*-axis. At the end of day 3 participants answered a similar free-recall questionnaire as in Experiment 1. It was supplemented by questions concerning the relationship between the tones and the tracking curve and by a visual forced-choice test.

#### Data Analysis

To test for learning, we submitted RMSEs to a Group × Block × Segment analysis of variance (ANOVA) as in Experiment 1. We also performed additional analyses for retention and transfer effects. To test for retention and transfer effects, we performed two Group × Block × Segment ANOVAs with three levels on Group, three levels on Block (retention: last group-practice block vs. group retention vs. single retention; transfer: last group-practice vs. *x*-transfer and *y*-transfer) and two levels on Segment. We used one-way ANOVAs or *t*-tests to investigate group effects where appropriate. Performance on the secondary task was assessed by calculating the percentage of correct responses to high-pitched and low-pitched tones and the reaction times to high-pitched and low-pitched tones. We submitted those to independent *t*-tests. Prior to testing the main hypothesis, we found that there were no significant differences between the three groups in age, gender, previous experience in tracking, motivation, or instruction. Significance level was set at *p* < 0.05, data were tested for distribution normality, and where appropriate degrees of freedom were adjusted for violations of sphericity using the Huynh–Feldt correction. Partial eta squared is reported (small effects $\eta_{p}^{2}$ > 0.01; medium effects $\eta_{p}^{2}$ > 0.06; large effects $\eta_{p}^{2}$ > 0.14).

### Results

#### Acquisition

**FIGURE 3:**
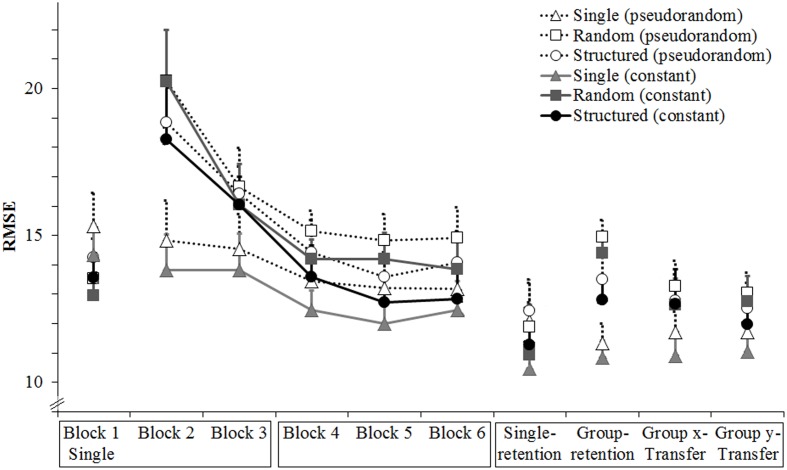
**Experiment 2: Mean RMSE over blocks, groups and segments.** Error bars are standard error of the mean. The *x*-labels are boxed according to the days when they were completed; Blocks 1–3 were completed in day 1, blocks 4–6 were completed in day 2, and the retention and transfer blocks were completed in day 3. Single denotes a block where all groups performed only the tracking task. Note that the axes are different from those used in **Figure 2**.

There was a significant main effect of block, *F*(1,27) = 53.41, *p* < 0.05, $\eta_{p}^{2}$ = 0.66, because mean error was smaller in block 6 than in block 2 (respectively *M* = 13.4, *SD* = 2.7 and *M* = 17.4, *SD* = 4.4). There was a significant main effect of segment, *F*(1,27) = 15.58, *p* < 0.05, $\eta_{p}^{2}$ = 0.37, because overall error was smaller on the constant than on the pseudorandom segment (respectively *M* = 14.8, *SD* = 2.7 and *M* = 16.0, *SD* = 3.8). There was a significant main effect of group, *F*(2,27) = 3.88, *p* < 0.05, $\eta_{p}^{2}$ = 0.22, because the Single group was significantly more accurate than the Random group (*p* < 0.05) but not significantly different from the Structure group (*p* > 0.05). There was a Group × Block interaction, *F*(1,27) = 5.87, *p* < 0.05, $\eta_{p}^{2}$ = 0.30, because from block 2 to 6 there was a larger improvement in the Random and Structure groups than in the Single group (respectively, differences of 5.9, 4.6, and 1.4; see **Figure 3**). There was a tendency for a Group × Block × Segment interaction, *F*(1,27) = 3.05, *p* = 0.05, $\eta_{p}^{2}$ = 0.18.

#### Retention

There was a significant effect of block, *F*(2,54) = 9.05, *p* < 0.05, $\eta_{p}^{2}$ = 0.25, because performance was significantly better in the single-retention block (*M* = 11.37, *SD* = 2.2) than in both the last practice (*M* = 13.42, *SD* = 3.7, *p* < 0.05) and the group-retention (*M* = 12.6, *SD* = 3.7; *p* < 0.05) blocks. There was a significant effect of segment, *F*(1,27) = 34.01, *p* < 0.05, $\eta_{p}^{2}$ = 0.56, because performance was better in the constant segment than in the pseudorandom segment (difference of 1.4). There was no group effect, *F*(2,27) = 7.98, *p* > 0.05, and no significant interactions.

#### Transfer

There was a significant effect of block, *F*(2,54) = 7.84, *p* < 0.05, $\eta_{p}^{2}$ = 0.23, because performance was marginally better in the last practice (*M* = 11.64, *SD* = 4.4) than in the *y*-transfer (*M* = 13.33, *SD* = 4.4; *p* = 0.07). There was a significant effect of segment, *F*(1,27) = 24.65, *p* < 0.05, $\eta_{p}^{2}$ = 0.48, because performance was better in the constant segment than in the pseudorandom segment (difference of 1.1). There was no group effect, *F*(2,27) = 1.74, *p* > 0.05, and no significant interactions.

#### Secondary Task Accuracy

**FIGURE 4:**
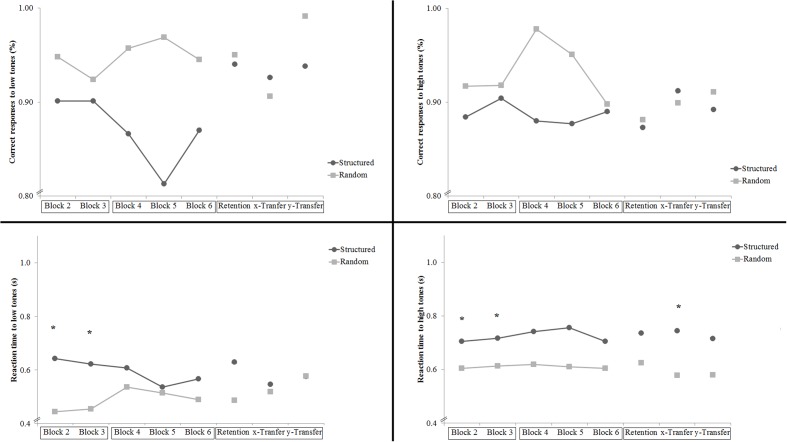
**Experiment 2: Performance measures in the secondary task.** Percentage of correct responses and reaction time to the low-pitched tones and the high-pitched tones. The *x*-labels are boxed according to the days when they were completed. Stars indicate significant group differences.

Participants in the Dual groups performed the secondary tasks well, with both groups in all blocks responding correctly to low-pitched and high-pitched tones between 81 and 99% of the time and taking between 0.45 and 0.76 s to respond. There were no significant group differences in the percentage of correct responses (see **Figure 4**). Group comparisons showed that the Random group reacted faster to low-pitched tones in blocks 2 and 3 [respectively, *t*(18) = 5.70, *p* < 0.001; *t*(18) = 4.03, *p* < 0.05]. The random group also reacted faster to high-pitched tones in blocks 2, 3, and *x*-transfer [respectively, *t*(18) = 2.94, *p* < 0.05; *t*(18) = 2.33, *p* < 0.05; *t*(18) = 2.29, *p* < 0.05].

#### Explicit Knowledge

Participants did not verbalize knowledge or awareness of the constant segment. Asked whether they realized there was one constant segment during the practice blocks, 17 participants answered that they did not realize anything, 13 participants answered that they did not notice it in the beginning but they thought that it could be possible (eight in the Single group, two in the Structure group and three in the Random group). When asked to decide which of the three segments was constant, six participants could not tell at all and did not answer, 10 guessed one of the outer segments, and 14 guessed correctly it was the middle (five in the Single group, two in the Structure group and three in the Random group).

### Discussion

Experiment 2 does not entirely confirm our hypothesis because the Structure group did not perform significantly different from the two other groups. To recall, Experiment 2 was designed to increase the difficulty of the secondary task (see **Table 2**) to test whether the beneficial effects of the Structured group that were observed in Experiment 1 would be retained. The increased difficulty was visible in the primary task where RMSEs were larger than in Experiment 1 in the first block of group-specific practice (difference of 5.6 between Experiments 1 and 2). The secondary tasks were different and therefore harder to compare between Experiments 1 and 2 but the percentage of correct responses to the high-pitched tones was higher in Experiment 1 (96% correct recall) than in Experiment 2 (90% correct foot taps). The costs associated with the increased difficulty of the secondary task seem to have been shared between the primary and secondary task.

The fact that the Structure group showed no advantage over the two other groups calls into question the beneficial effects of a shared structure between the primary and secondary task. At the same time, the Random group was not hampered significantly in their performance. It may be that because performance was quite accurate in all groups, there was no reason to exploit further optimization strategies. This is in line with a previous study which found that participants will learn to exploit additional relevant information in a tracking task only if other relevant information is withdrawn (Raab et al., 2013). In other words, the optimization of strategies may only come about under more stringent conditions. With this in mind we designed a third experiment where we increased the motor load of the primary task while maintaining other parameters similar to Experiment 2. We expected that under more demanding conditions the Structure group would exploit the structure of the primary and secondary task to improve their performance relative to the Random group.

## Experiment 3

The goal of the third experiment was to further test the robustness of task integration by increasing the motor load of the primary tracking task. Tracking was performed faster than in the previous experiments and with the non-dominant hand. We asked participants to perform a manual tracking task while tapping a foot on high-pitched tones and tapping another foot on low-pitched tones as in Experiment 2. The manipulation was also the same: a Single group performed the tracking task only, a Random group for who the low-pitched tones were presented rhythmically and the high-pitched tones were presented randomly, and a Structure group for whom the low-pitched tones were also presented rhythmically but the high-pitched tones were temporally coupled to the tracking task. We hypothesized that the Structure group would out-perform the Random group because the structure of the secondary task allows for task integration in implicit motor learning.

### Method

#### Participants

Participants were 30 university students (16 female and 14 male; *M* = 24.57 years, *SD* = 3.92). They had normal or corrected-to-normal vision and normal hearing abilities and had no prior experience in tracking. Participants received course credit and provided informed consent before starting the experiment. Participants were randomly assigned to one of the three groups ensuring a near-equal distribution of gender and handedness.

#### Apparatus and Material

There were only two differences between this and the previous experiment, both aimed at increasing the load of the primary task. The speed was faster with trial duration 25 s (instead of the previous 35 s) and participants used their non-dominant hand to perform the primary tracking task.

#### Design and Procedure

We used a factorial design with three factors: Group × Block × Segment. The groups were: Single, who only performed the primary tracking task; Random, who responded to low- and high-pitched tones presented randomly; Structure, who responded to randomly presented low-pitched tones and high-pitched tones presented 250 ms before each curve.

Participants reported to the laboratory for the experimental sessions on three consecutive days. They were randomly assigned to one of the three groups that received either the single task (Single) or one of the dual tasks (Structure, Random). Instructions and procedure were similar to Experiment 2 but tracking was done with the non-dominant hand at a higher speed. On Day 1 participants completed one block of 40 trials in single-task as a baseline. Afterwards they did 10 trials with a random middle segment in single-task as a catch block to test the learning of the constant segment. The following block of 40 trials was performed under group-specific conditions. On Day 2 participants did one practice block of 40 trials in group-specific conditions followed by a catch block of 10 trials in single-task and another group-specific practice block of 40 trials for a total of 180 trials over 2 days. On day 3, participants completed a retention test of 10 trials in single-task, followed by another 10 retention trials in their group-specific condition. This was followed by two transfer tasks of 10 trials each, with the middle segment reversed along the *x*-axis or the *y*-axis. At the end of Day 3 participants answered a free-recall questionnaire with the same questions as in Experiment 2 (see **Table 2**).

#### Data Analysis

To test for learning, we submitted RMSEs to a Group × Block × Segment analysis of variance (ANOVA). To test for block we used the first and last group-specific practice blocks 3 and 6. We also performed additional analyses for retention and transfer effects. To test for retention and transfer effects, we used two Group × Block × Segment ANOVAs with three levels on Group, three levels on Block (retention: last group-practice block vs. group retention vs. single retention; transfer: last group-practice vs. transfer-*x* and transfer-*y*), and two levels on Segment as in Experiment 2. To test the catch trials we used a Group × Block × Catch × Segment ANOVA with three levels on Group, two levels on Block (start vs. end of practice), two levels on Catch (normal with a constant middle segment vs. catch with a random middle segment), and two levels on Segment. We used one-way ANOVAs or *t*-tests to investigate group effects where appropriate. Performance on the secondary task was assessed by calculating the percentage of correct responses to high-pitched and low-pitched tones as well as reaction times to high-pitched and low-pitched tones. We tested for group effects using independent *t*-tests. Prior to testing the main hypothesis, we found that there were no significant differences between the three groups in age, gender, previous experience in tracking, motivation, or instruction. Significance level was set at *p* < 0.05, data were tested for distribution normality, and where appropriate degrees of freedom were adjusted for violations of sphericity using the Huynh–Feldt correction. Partial eta squared is reported with small ($\eta_{p}^{2}$ > 0.01), medium ($\eta_{p}^{2}$ > 0.06) and large ($\eta_{p}^{2}$ > 0.14) effect sizes.

### Results

#### Acquisition

**FIGURE 5:**
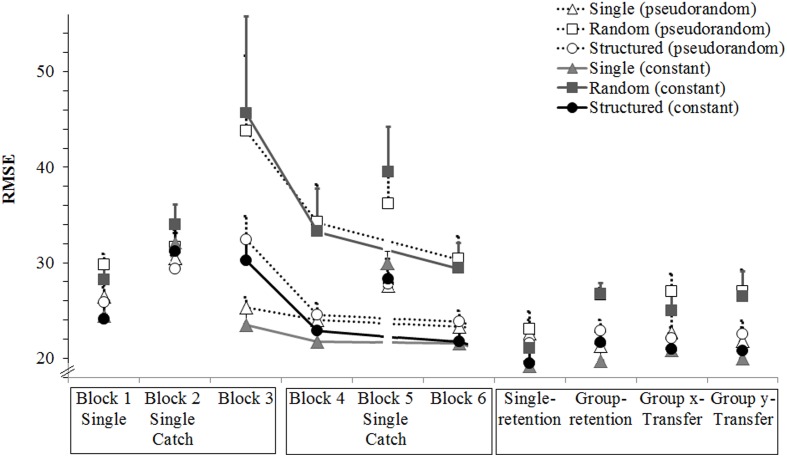
**Experiment 3: Mean RMSE over blocks, groups and segments.** Error bars are standard error of the mean. The *x*-labels are boxed according to the days when they were completed; Blocks 1–3 were completed in day 1, blocks 4–6 were completed in day 2, and the retention and transfer blocks were completed in day 3. Single denotes a block where all groups performed only the tracking task. Catch denotes a block where all groups performed only the tracking task and all segments were pseudorandom. Note that group practice blocks 3, 4, and 6 are linked to facilitate comparison but a 10-trial catch block was completed between blocks 4 and 6. In total there were three blocks of 40 group practice trials (fewer than in the previous experiments). Note that the axes are different from those used in previous figures.

There was a significant main effect of block, *F*(1,27) = 37.64, *p* < 0.05, $\eta_{p}^{2}$ = 0.58, because mean error was smaller in block 6 than in block 3 (respectively *M* = 24.7, *SD* = 3.8 and *M* = 31.8, *SD* = 8.8). There was a tendency for an effect of segment, *F*(1,27) = 3.98, *p* = 0.056, $\eta_{p}^{2}$ = 0.13, because mean error was smaller on the constant than the pseudorandom segment (respectively *M* = 26.7, *SD* = 3.8 and *M* = 29.9, *SD* = 9.9). There was a significant main effect of group, *F*(2,27) = 8.33, *p* < 0.05, $\eta_{p}^{2}$ = 0.38, because the Random group performed significantly worse than the Single and the Structure groups (*p*s < 0.05). Importantly, there was a significant Group × Block effect, *F*(2,27) = 5.12, *p* < 0.05, $\eta_{p}^{2}$ = 0.28, because the Structure group performed worse than the Single group in block 3 (*p* < 0.05; *M* = 31.3, *SD* = 15.3 vs. *M* = 24.4, *SD* = 15.3) but performance was similar in block 6 (*p* > 0.05; *M* = 22.8, *SD* = 7.1 vs. *M* = 22.4, *SD* = 7.1; see **Figure 5**).

#### Retention

There was a significant effect of block, *F*(2,54) = 13.93, *p* < 0.05, $\eta_{p}^{2}$ = 0.34, because single-retention was better than group-retention which was better than last practice (*p*s < 0.05). There was a significant effect of segment, *F*(1,27) = 30.86, *p* < 0.05, $\eta_{p}^{2}$ = 0.53, with better performance in the constant segment (difference of 2.1). There was a significant effect of group, *F*(2,27) = 4.12, *p* < 0.05, $\eta_{p}^{2}$ = 0.23, because the Random group (*M* = 25.6, *SD* = 6.6) performed significantly worse than the single (*M* = 21.3, *SD* = 6.6; *p* < 0.05) and structured (*M* = 21.9, *SD* = 6.6; *p* < 0.05) groups. There was also a significant Group × Block effect, *F*(4,54) = 3.76, *p* < 0.05, $\eta_{p}^{2}$ = 0.22. A planned oneway ANOVA revealed that, in both the constant and random segments, the Random group performed worse than the other two groups at the last practice (*p*s < 0.05), and worse than the single group in the group-retention (*p*s < 0.05).

#### Transfer

There was a significant effect of block, *F*(2,54) = 9.98, *p* < 0.05, $\eta_{p}^{2}$ = 0.27, because performance in the last practice was worse than in both transfer blocks (*p*s < 0.05). There was a significant effect of segment, *F*(1,27) = 16.28, *p* < 0.05, $\eta_{p}^{2}$ = 0.38, with better performance in the constant segment (difference of 2.2). There was a significant effect of group, *F*(2,27) = 4.47, *p* < 0.05, $\eta_{p}^{2}$ = 0.25, because the Random group (*M* = 26.6, *SD* = 7.1) performed significantly worse than the single (*M* = 21.7, *SD* = 7.1; *p* < 0.05) and structured (*M* = 22.0, *SD* = 7.1; *p* < 0.05) groups. There were no significant interactions.

#### Catch Trials

There was a significant main effect of the catch, *F*(1,27) = 169.84, *p* < 0.05, $\eta_{p}^{2}$ = 0.86, and a significant Catch × Segment interaction, *F*(1,27) = 134.79, *p* < 0.05, $\eta_{p}^{2}$ = 0.83. This was because on catch trials participants performed better on the pseudorandom segments than on the middle segment (which usually was constant but was random in the catch trials), whereas on normal trials participants performed better on the middle (constant) segment. There were also significant effects of group, *F*(1,27) = 5.09, *p* < 0.05, $\eta_{p}^{2}$ = 0.27, and a Group × Block interaction, *F*(1,27) = 4.04, *p* < 0.05, $\eta_{p}^{2}$ = 0.23. As expected the Random group performed worse than the Single and Structure groups (*p*s < 0.05).

#### Secondary Task Accuracy

**FIGURE 6:**
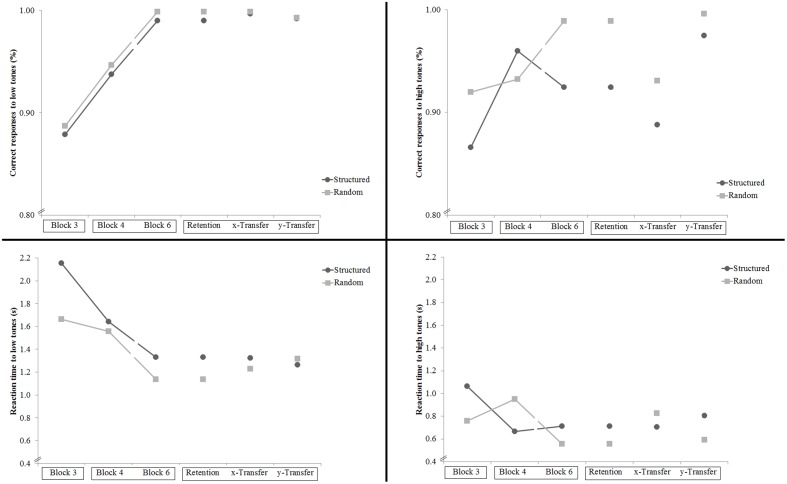
**Experiment 3: Percentage of correct responses and reaction time to the low-pitched tones and the high-pitched tones in the secondary tasks.** Interruptions in the line between Blocks 4 and 6 indicate that a 10-trial catch block was completed between the two blocks. The *x*-labels are boxed according to the days when they were completed. There were no significant group differences.

Participants in the Dual groups performed the secondary tasks well, with both groups in all blocks responding correctly to low-pitched and high-pitched tones between 88 and 100% of the time and taking between 0.56 and 2.16 s to respond. There were no significant group differences in the percentage of correct responses or in the reaction times to high-pitch or low-pitched tones (see **Figure 6**).

#### Explicit Knowledge

Participants did not verbalize knowledge or awareness of the constant middle segment. Asked whether they realized there was one constant segment during the practice blocks, 24 participants answered that they did not realize anything, six participants answered that they did not notice it but they thought that it could be possible (three in the Single group, one in the Structure group and two in the Random group). When asked to decide which of the three segments was constant, 20 participants could not tell at all and did not answer, three guessed it was one of the outer segments, and seven guessed correctly the middle segment (three in the Single group, two in the Structure group and two in the Random group).

### Discussion

Experiment 3 confirms our hypothesis that the Structure group out-performed the Random group. To recall, Experiment 3 was designed to increase the difficulty of the primary task (see **Table 2**). The rationale was that a harder task may provide the incentive to exploit the information available that can benefit performance – in this case exploit the structure shared between primary and secondary task. The increased difficulty was visible in the primary task where RMSE were larger than in Experiment 2 in the first block of group-specific practice (difference of 18.6 between Experiments 2 and 3). The secondary task, which was the same as in Experiment 2, also suffered. Both the percentage of correct responses and reaction times to both high-pitched and low-pitched tones were worse in Experiment 3 than 2. Unlike Experiment 2, the costs associated with the increased difficulty of the primary task seem to have impacted only the primary task.

The presence of implicit learning was indicated by the main effect of segment during acquisition, retention, transfer, its interaction with the catch blocks, and the lack of explicit knowledge. However, there was no evidence for a beneficial effect of the Structured group in implicit learning because we found no significant interaction between Group and Segment and no group differences in the single-retention block.

We found a strong beneficial effect of the shared structure. All participants were presented with a new path on every trial and therefore the tones never occurred at the same time; for the Structured group, however, high-pitched tones occurred at the same time in relation to the curve therefore these tones and the path shared the same temporal structure. Note that these tones were not otherwise more regular or consistent than those in the random group. Therefore if the Structured group performed better than the Random group it was because they were able to use this structure to perform the tracking task.

## General Discussion

The aim of this study was to investigate task integration in implicit motor learning. The results show that a similar structure between the primary and the secondary tasks can be beneficial in multitasking. The structure that existed between the primary and secondary tasks was the only difference between the multi-task groups, yet their performance was different. This means that the detrimental effects of multi-tasking were lessened when participants could use the structural similarity between primary and secondary tasks (e.g., Schmidtke and Heuer, 1997; Ruthruff et al., 2006). To our knowledge this is the first time that the benefits of dual-task performance have been tested directly by manipulating the characteristics of the dual-task. However, recent studies have argued for the protective effect of other bottom-up processes in dual tasks. For instance, Cutanda et al. (2015) showed that regular rhythms can enhance temporal preparation under dual-task conditions involving working memory. Huestegge and Koch (2014) showed that dual-task decrements can be averted if tasks are usually coupled together. In their study they showed peripheral stimuli and asked participants to either saccade, press a key, or do both. They found that participants performed significantly better when the task involved both saccading and manual pressing. Because these two actions are usually coupled together, the authors propose that the degree of saccade automaticity is essential for this dual-task benefit. Our study differs from those of Huestegge and Koch because our tasks did not involve an action that was previously automatically coupled with another action but our results are similar and may rely on a similar mechanism. After little practice the participants in the Structure group benefitted from the shared structure between the high-pitched tones and the curves in the path.

In our first experiment, the group performing under the structure condition outperformed the group in the single-tracking task. This means that the structure of the task not only obliterated the detrimental effects of the dual-task but also that the tone-counting task aided the performance of the tracking task. Another study by Jiang and Swallow (2014) also found that the structure of the task can obliterate the detrimental effects of dual-tasking. They presented male or female faces and high or low tones which could be target(s) or non-target(s). Participants performed better in a recognition test when both face and tone had been presented congruently than when they were presented incongruently. The authors propose that the attentional selection for separate tasks occurred at the same time and that this dual-task benefitted recall. Our task did not involve recall in the same way but the temporal presentation of our stimuli was congruent for the structure group. Therefore these results extend those of Jiang and Swallow (2014) regarding congruency as a potential aid to dual-tasking performance.

In our second and third experiments the motor load of the tasks increased (note the different scales in the figures axes across experiments). When the difficulty increased in the secondary task, the performance on the primary task was better for the single group but not statistically different between the two dual-task groups. However, when the difficulty increased also in the primary task, the performance of the Structure group was level with that of the Single group and significantly better than the Random group. In hindsight, the difficulty of the secondary task may have been harder for the Structure group because it involved a rule for each foot whether the Random group had the same rule for both feet. Regardless, our results are in accordance with a previous tracking study (Raab et al., 2013), which found that participants only learned to exploit the information available when they were forced to do it in order to meet performance demands. We propose that the difficulty of the tasks in the last experiment invited participants to exploit and use the structure of the task to cope with its demands. This will have in turn facilitated their performance at both retention and transfer where they systematically outperformed the Random group.

Although unrelated with the effect of structure, this study also shows strong effects of implicit learning that are consistent throughout the three experiments. Implicit learning was evidenced by better performance in the constant middle segment than on the outer pseudorandom segments. This effect was visible during learning, at retention, at transfer and also on the catch trials used in the last experiment. Moreover, although the characteristics of the constant segment were learned, participants reported no explicit knowledge of the pattern or its repeatability. This is in agreement with previous research, for instance by Magill (1998), Wulf (2007), and Zhu et al. (2013) who also found implicit learning of different patterns.

We found beneficial effects of structure on performance but not on implicit learning. The task integration therefore served the purpose of solving the multi-task problem at hand but it did not extend further into a learned pattern. It is possible that task integration can still benefit implicit learning if more practice time and/or more capacity is available. Both the findings of Heuer and Schmidtke (1996) and ours add evidence in favor of a systematic relation of attention capacity and task difficulty in visual search paradigms in general, indicating that effects are determined by the nature of the tasks (Huang and Pashler, 2005). Our results show performance decrements with increased load visible between experiments. Changes in the difficulty of the learning task can be realized in different ways (e.g., Ruthruff et al., 2006), but it is plausible that the manipulation of sensory and motor systems will affect specific components of both primary and secondary task performance (Mayr, 1996). Also, the potential integration may depend on the sensorimotor interfaces which are possible between tasks. We have studied integration in terms of the temporal relationship between the primary and secondary tasks but other types of integration might be explored as well. For instance, the predictability or automaticity of a task may also aid multi-task performance (Medeiros-Ward et al., 2014; Cutanda et al., 2015; Fischer and Dreisbach, 2015) but the conditions for these effects and the mechanisms underlying potential effects need further research.

## Conclusion

Alongside the detrimental effects of multi-tasking it is important to investigate which task characteristics may enhance performance. Here we found examples where the temporal characteristics of the primary and secondary tasks considerably aided multi-task even under stringent sensory-motor load. Understanding these characteristics better will enable the creation of multi-task schedules which benefit performance and may provide a better data-led understanding of the mechanisms underlying perceptual-motor control.

## Ethics Statement

Ethics committee for the Institute for Sport and Sport Science of the University of Heidelberg, Germany. Participants who were informed about the study and agreed to participate voluntarily, were asked to read and sign and informed consent sheet before the experiment started.

## Author Contributions

All authors contributed to writing; MR, MH, and JS contributed to data collection; RO contributed to data analysis and results.

## Conflict of Interest Statement

The authors declare that the research was conducted in the absence of any commercial or financial relationships that could be construed as a potential conflict of interest.
